# The interface between dentistry and respiratory sleep disorders in children

**DOI:** 10.5935/1984-0063.20200004

**Published:** 2020

**Authors:** Ricardo Leão Castilho, Lucas Hideki Matsumoto, Gustavo Leão Castilho, Silke Anna Theresa Weber

**Affiliations:** Botucatu Medical School, Otorhinolaryngology and Head and Neck Surgery - Botucatu - SP - Brazil.

**Keywords:** Mouth Breathing, Sleep Bruxism, Dental Caries, Malocclusion

## Abstract

**Objective:**

To study the incidence of mouth breathing and its association with sleep disorders, dental caries, malocclusion and deleterious oral habits, in children.

**Material and Methods:**

152 children (6 to 9 years), of both genders, were invited to perform clinical evaluation of the oral cavity and the application of the OSA-18.

**Results:**

89 presented mouth breathing (MB), being 45% with malocclusion, 56% with dental caries, 38% with tooth loss, 51% with bruxism and 52% with the habit of sucking ﬁnger or paciﬁer, compared to 40%, 40%, 21%, 27% and 43%, respectively, in the 63 children with nasal breathing (NB). 35 MB showed moderate to high risk for OSAS, while only 8 of the children showed moderate risk. The average score of OSA-18 was 50 (MB:57/NB:40), with 29 (19%) children showing moderate risk. Among these, 74% presented mouth breathing, 26% malocclusion, 61% dental caries, 35% tooth loss, 42% bruxism and 55% sucked ﬁnger or paciﬁer, and in the 14 (9%) with high risk, they were 100%, 75%, 58%, 50%, 67% and 67%, respectively.

**Conclusion:**

High frequencies of respiratory disorders with sleep repercussions associated with oral alterations were observed, reinforcing the correlation between mouth breathing and changes in stomatognathic system.

## INTRODUCTION

Sleep disorders are a problem of growing importance in pediatrics. In addition to snoring, it encompasses more serious disorders, such as obstructive sleep apnea syndrome (OSAS). As the number of individuals with a respiratory disorder is increasing, OSAS can be considered a public health problem that deserves greater attention in relation to the diagnoses and treatments being performed. Pediatric OSAS differs from adult OSAS and in some cases can be a serious medical condition with unique characteristics, diagnosis, and management recommendations^1^. Often the signs and symptoms of OSAS in the child are not properly identified by healthcare providers and practitioners, something that will not only affect the immediate well-being of the child, but will have adverse effects on the health of long-term patients as adults.

Sleep disorders affect children’s quality of life and are related to mouth breathing, a major cause of malocclusion. Alterations in saliva-mediated defense mechanisms and deleterious oral habits may be associated with caries, changes in tooth eruption and development of the jaws, respectively. The objective of this research was to study the incidence of mouth breathing and its association with sleep disorders, dental caries, tooth loss, malocclusion and deleterious oral habits in preschool children.

## MATERIAL AND METHODS

### Study design

The research, sent and approved by the Ethics Committee in Research on Human Beings (959.505), was carried out in a transversal way, from three schools with different socioeconomic indicators, chosen by the Education Department of the Municipality of Botucatu/SP, Brazil. All children were informed of free and informed consent (FIC) to be signed by those responsible and those who agreed to participate in the study were invited to perform a clinical evaluation of the child’s oral cavity and answered the questionnaires OSA-18 and of habits by its responsible, during the period of May 2016 to November 2017.

As a risk assessment for OSAS, the OSA-18 questionnaire was used, consisting of 18 items grouped into 5 domains, whose items are scored on an ordinal scale of 7 points (1 - no time; 2 - almost no time; 3 - a few times; 4 - sometimes; 5 - several times; 6 - most times; 7 - every time). Thus, the OSA-18 domains can obtain the following scores:

a) Sleep disorder (4 items with scores varying from 4 to 28);
b) Physical suffering (4 items with scores varying from 4 to 28);
c) Emotional distress (3 items with scores varying from 3 to 21);
d) Diurnal problems (3 items with scores varying from 3 to 21);
e) Concerns of parents or guardians (4 items with scores varying from 4 to 28).

The result of the OSA-18 questionnaire is given by the sum of the values chosen by the person interviewed for the frequency with which the cited events occur. The numerical value can be translated as low (<60 points), moderate (≥60 points and <80 points) or high (≥80 points) risk for OSAS by the child^2,3^.

The evaluation form assesses whether occlusion is favorable, classification of malocclusion, occurrence of dental loss, overbite and crossbite, as well as whether preventive intervention and interception were performed. Data collection was performed by oral clinical examination and a questionnaire directed to parents, lasting approximately 15 minutes. It is noteworthy that the American Academy of Sleep Medicine (AASM) considers that reports from parents/guardians are quite reliable and sufficiently objective for use in epidemiological studies^4^.

### Sample size, selection and inclusion criteria

According to demographic data of the city of Botucatu (IBGE) of 2009, there are 9,888 children in the age group of 5 to 9 years. Considering the estimated frequency of respiratory disorders of 10% of the pediatric population, the estimated number would be 988.

Five schools were invited to participate in the research, with only three participating effectively. In total, we had access to 750 children, of whom 417 agreed to participate and returned the completed and signed FIC. 199 children attended the evaluation, divided into 170 with complete data according to the proposed protocol, and 29 with incomplete data. Among the 170 children, aged between 6 and 11 years, 152 were considered, with ages ranging from 6 to 9 years, age range with largest casuistic in literature on the topic studied.

Considering the number of children in the public school system N=750, the sample size was calculated, with a prevalence of 50%, a margin of error of 7% and a 95% confidence, obtaining an N=156 corrected by the population finite.

It is worth mentioning that numerous attempts have been made to count on the participation of those responsible for meetings, including some children were previously evaluated, with the expected completion of the questionnaires, which did not materialize.

### Study variables

The quantitative variables age and outcome of the OSA-18 questionnaire were evaluated, besides the qualitative variables gender, mouth breathing, malocclusion, dental caries, tooth loss, bruxism and finger/pacifier suction.

### Statistical analysis

A descriptive statistical analysis of the data with frequency and percentage for the qualitative variables, and mean, median, standard deviation, minimum and maximum values for the quantitative variables were performed on 01/22/2018.

Significant variables at 95% were analyzed when related to high risk to OSAS and mouth breathing. To verify the association of OSA-18 and dental disorders a Chi-square or Fisher’s exact test was performed when necessary. As for age, a Test of Normality of the data was performed and as they had a symmetrical distribution, ANOVA followed by Tukey was used for the multiple comparisons. *P*<0.05 was considered as level of significance. The program used to perform the analysis was the SAS system, version 9.3.

## RESULTS

The distribution of the children interviewed according to sex and age is shown in Graph 1. No significant statistical difference was found in the results of the OSA-18 questionnaire for moderate and high risk between genders and age groups, but was found for malocclusion.

**Graphic 1:**
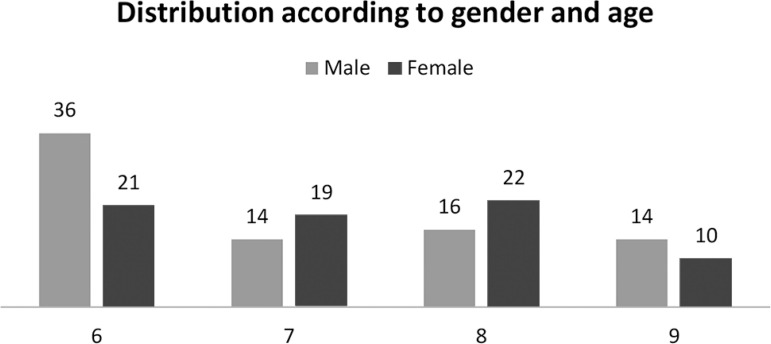
Distribution of 152 children evaluated according to gender (male / female) and age (6-9 years).

Of the 152, 89 (59%) presented mouth breathing (MB). Of these, 40 (45%) had malocclusion, 50 (56%) had dental caries, 34 (38%) tooth loss, 45 (51%) bruxism and 46 (52%) sucked finger or pacifier, compared to 40%, 40%, 21%, 27% and 43%, respectively, in the 63 children with nasal breathing (NB) (Table 1). 35 MB (39%) showed moderate to high risk for developing OSAS, while only 8 of the NB (13%) showed moderate risk.

**Table 1:** Values of dental disorders present in 152 children, divided into mouth breathers (MB) and nasal breathers (NB), with the OSA-18 means of each group.

n=152	MB (n=89)	NB (n=63)	*p*
Malocclusion	40 (45%)*	25 (40%)	0.5183
Dental Caries	50 (56%)	25 (40%)	0.0451*
Tooth Loss	34 (38%)	13 (21%)	0.0210*
Bruxism	45 (51%)	17 (27%)	0.0036*
Suction Finger/Pacifier	46 (52%)	27 (43%)	0.2832
OSA-18	57	40	

* Significant values p<0.05

The mean OSA-18 was 50 (MB: 57/NB: 40). 109 (72%) children had a low risk for OSAS (OSA-1) and among these, 54 (50%) had mouth breathing, 48 (44%) had malocclusion, 49 (45%) dental caries, 30 (28%) tooth loss, 41 (38%) bruxism and 48 (44%) sucked finger or pacifier. In 29 (19%) patients with moderate risk for OSAS (OSA-2), the proportions were 74%, 26%, 61%, 35%, 42% and 55%, and in 14 (9%) patients with a high risk for OSAS (OSA-3), were 100%, 75%, 58%, 50%, 67% and 67%, respectively (Table 2).

**Table 2:** The values of odontologic disorders present in 152 children, distributed according to the risk of OSAS, classified as OSA-1 (low), OSA-2 (moderate) and OSA-3 (high), with OSA-18 averages of each group.

n=152	OSA-1 + OSA-2	OSA-3	*p*
	138	14	
Mouth Breathing	77 (56%)	12 (100%)	0.0010*
Malocclusion	56 (41%)	9 (75%)	0.0876
Dental Caries	68 (49%)	7 (58%)	0.2405
Tooth Loss	41 (30%)	6 (50%)	0.1050
Bruxism	54 (39%)	8 (67%)	0.0144*
Suction Finger/Pacifier	65 (47%)	8 (67%)	0.4737
OSA-18	45	92	

* Significant values p<0.05

## DISCUSSION

In recent years, numerous studies have examined sleep disorders in the context of different odontological findings in a variety of age groups. The findings of the presented study, in agreement with the literature in some respects, while disagreeing in others, demonstrate a high interindividual variability of the breathing pattern^5,6^. The key question was the number of children in this sample who presented mouth breathing associated with sleep disorders, dental caries, malocclusion and deleterious oral habits, and the response indicates a high incidence.

In total, 417 terms of free and informed consent were signed by the parents or guardians of the students of the three schools evaluated. Only 170 (41%) actually participated in the evaluation, while 218 (52%) did not attend the calls and 29 (7%) provided incomplete data. This reality denotes the difficulty of raising the public’s awareness in a general way in the diagnosis and treatment of health problems of their respective children in the school environment.

The presence of mouth breathing was significant for a high risk for OSAS, something expected because it is one of the classic OSAS symptoms^7^. OSA-18 mean score for nasal breathers was 40 points, while for mouth breathers it was 57, close to the cut-off point for moderate risk of OSAS, demonstrating the importance of respiratory pattern in pathophysiology of OSAS^3^. Similarly, bruxism, a parasomnia frequently associated with OSAS, was a significant finding in children at higher risk, corroborating findings from the literature^8,9^.

A high prevalence of mouth breathers was observed in our sample (59%), similar to other studies, with no difference between the genders, confirming what is reported in the literature between the ages of 2 and 18 years^6,10,11^. Cited data act as an alert to society, which is facing a real public health problem. The lack of investigation of respiratory disorders such as mouth breathing, snoring and even risk to OSAS shows that the dissemination, training and interaction of professionals in the area of Dentistry with Sleep Medicine has been ineffective.

The presence of malocclusion was considered significant, requiring greater care when evaluating the child’s oral condition, considering the possibility of being a mouth breather or presenting signs of OSAS. Recent data in the literature indicated that orthodontic and dentofacial orthopedic treatment may decrease obstructive respiratory events in some pediatric patients with OSAS^12^. The municipality of Botucatu has a program with dental professionals working in public schools, which has shown low effectiveness in the control and promotion of oral health.

This demonstrates the need to raise awareness of the benefits of maintaining good oral health so that this knowledge can be transformed into positive attitudes, thus minimizing the suffering of these children. There is a need for greater clarification on the part of dentists about the care related to these patients, mainly in order to improve access to dental treatment.

Health is the product of interaction with the family, culture, social structure and physical development. Oral health promotion actions aimed at early childhood should prioritize the education of parents, helping to build healthy habits that will reduce the occurrence of diseases and improve the oral health of the whole family^13^.

## CONCLUSION

Significant evidence of respiratory problems, including sleep disorders, associated with oral alterations, has been observed suggesting a considerable relation between mouth breathing in children and changes in stomatognathic system.
